# Psychosocial interventions for improving the physical health of young people and adults with attention deficit hyperactivity disorder: a scoping review

**DOI:** 10.1186/s12888-024-06009-2

**Published:** 2024-08-20

**Authors:** John Headley Ward, Audrey McBride, Anna Price, Tamsin Newlove Delgado

**Affiliations:** 1 2.05 South Cloisters, 2.05 South Cloisters, St Luke’s Campus, University of Exeter Medical School, Heavitree Road, Exeter, EX2 4TH UK; 2 Royal Devon University NHS Foundation Trust, Exeter, UK; 3 Department of Psychiatry, University of Oxford, Oxford, UK; 4 Oxford Health NHS Foundation Trust, Oxford, UK

**Keywords:** ADHD, Preventative medicine, Sleep, Psychosocial intervention

## Abstract

**Background:**

Young people and adults with ADHD are at risk of a range of physical health problems. There is limited guidance on how to approach health problems in ADHD, and especially around 16-25 year olds who will be transitioning from paediatric to adult care. The aim of this scoping review was to identify psychosocial interventions that target physical health in young people and adults with ADHD.

**Methods:**

We constructed searches in MEDLINE, PsycInfo, EMBASE of adolescents, young people and adults. Inclusion criteria were; studies of psychosocial interventions examining a component of physical health, applicable to people aged 16-25, with clinical or research diagnoses of ADHD. Data were extracted using a data extraction tool and tabulated, including study intervention framing/aims, population, intervention, and relevant outcomes (including specific statistics where relevant).

**Results:**

Our search identified 22 unique papers covering, psychosocial interventions targeting at least one of sleep (*n=*7), smoking (*n=*3), substance/alcohol use (*n=*4), physical health/exercise (*n=*6) and general health (*n=*3). Studies examined psychotherapy/behaviour interventions (*n=*12), psychoeducation (*n=*4), digital (*n=*2) and social interventions (*n=*4). There was significant heterogeneity in intervention framing, outcome measures and population.

**Conclusion:**

Further work on the impact of targeted physical health interventions, with explicit reference to a conceptual framework of poor health in ADHD is required. Furthermore, future work standardising reporting of physical health outcomes in ADHD is crucial for the development of an evidence base in this field.

**Supplementary Information:**

The online version contains supplementary material available at 10.1186/s12888-024-06009-2.

## Background

Attention deficit hyperactivity disorder (ADHD) is a neurodevelopmental condition characterised by combinations of hyperactivity, impulsivity, and inattention, thought to affect 5.6% of 12-18 year olds and 2.58% of adults [1, 2]. Whilst traditionally thought of as a disorder of childhood, with a typical onset before the age of 12, it is now understood that symptoms can persist into adulthood and have a significant impact on many aspects of life, including physical health [3–6].

In respect to the links in research between ADHD and physical health, firstly there is a wealth of literature on the association between ADHD and higher rates of health risk behaviours, including smoking, alcohol abuse, substance misuse, risk-taking behaviour, self-harm, obesity and sleep disorders [6–11]. These findings are reproduced in studies using various methods including traits-based approaches, mendelian randomisation, case-control and longitudinal follow-up studies.

Secondly, there is a growing body of evidence demonstrating links between ADHD and non-communicable diseases. A large genetically informed Swedish registry study found that participants with ADHD were at higher risk for 34 out the 35 conditions studied compared to those without ADHD, including nervous system and respiratory disorders [12]. Other studies have also demonstrated high rates of neurological and respiratory disease, as well as gastrointestinal disorders and cardiovascular disease [13–15]. Furthermore, Stickley and colleagues [15] demonstrated that multimorbidity was predictive of whether study participants had ADHD. In respect to mortality, several longitudinal studies have noted increased mortality rates amongst people with ADHD. Whilst these appear to be driven by accidental and unnatural deaths, the cause remains contested [6, 16–18].

There have been various attempts to explain the inequalities in physical health outcomes for this population. It has previously been suggested that people with ADHD may be less likely to follow government recommendations on health promotion, even when controlling for socioeconomic status [19]. This is echoed in work by Cherkasova et al*,* who reported that the persistence of ADHD symptoms into adulthood mediated poorer functional outcomes [6]. However, the large sibling analysis study of DuRietz *et al* highlights the importance of genetic risk factors in the association between ADHD and physical health, supported by their finding that shared genetic factors explained 60-69% of the relationship between ADHD and respiratory, musculoskeletal and metabolic disorders in their sample [20].

There have been previous studies suggesting that some of the health risks in ADHD may be mitigated by appropriate treatment of ADHD using medication (e.g., meta-analyses demonstrating the efficacy of medication in improving sleep or substance misuse [7, 21, 22]). In addition to medication, psychosocial interventions are likely to be important in the prevention and mitigation of health risks in ADHD, when provided as part of a holistic approach. Importantly, psychosocial interventions can also constitute health promotion and support health autonomy, which may be of particular significance to young adults transitioning to adult care [23–27]. There is a small and heterogenous body of research examining the efficacy of psychosocial interventions in the management of physical health problems associated with ADHD [28–33]. However, this has not yet been synthesised to identify the nature and extent of existing research or indicate targets for future research and intervention development. This is a significant evidence gap given the poorer physical health of people with ADHD, which adversely affects quality of life and economic, social and health outcomes [34–37].

This scoping review aims to identify and describe existing psychosocial interventions for physical health in young people and adults with ADHD, including those in preliminary stages (e.g. feasibility trials).

## Methods

Given the lack of previous reviews in this field, and the need to provide a broad overview of available evidence, a scoping review was chosen to identify psychosocial interventions addressing physical health in ADHD. Scoping reviews are suitable for identifying research gaps, summarising research findings, clarifying concepts, and making recommendations for future research [38]. This scoping review aimed to identify relevant literature using an inclusive approach incorporating different methodologies and reflecting varying levels of quality [39].

The review followed a five-stage process as described by Arksey and O'Malley: identifying the question, identifying relevant studies, study selection, charting the data and collating, summarising and reporting the results [40, 41]. We found no previous scoping reviews or systematic reviews examining this topic. We have reported our scoping review according to the Preferred Reporting Items for Systematic Reviews and Meta-Analyses Scoping Review Extension checklist (see supplementary materials) [42]. We did not pre-register this review protocol.

### Eligibility criteria

Studies were eligible for inclusion in the review if they met four key eligibility criteria: a population aged 16 years or older (or including substantial representation of this group), diagnosed with ADHD (either clinically diagnosed, self-reported, or using a standardised diagnostic measure) (population and context), the introduction of a form of structured psychosocial intervention within an experimental trial (concept) and the measurement of a physical health outcome (outcome).

In respect to defining the population of interest, we sought interventions that would be relevant to young people and adults, of a transition care age group (16-25 year olds). This age bracket is important for two reasons. Firstly, it is well-understood that ADHD care begins to decline for older adolescents, as medication adherence and service access decline [25, 26]. Secondly, the transition to adulthood is when adolescents begin to take responsibility for their own health and health behaviour, and therefore support around independent health management would be timely [23, 24, 27].

To meet the scoping review’s aim of identifying all interventions relevant to the population, we included studies of under 16s, and some studies of adults over 25 years old. This was the case where the proposed intervention had methods or findings that were clearly applicable to 16–25-year-olds. ‘Applicable’ in this context was defined as interventions that could be utilised in our population of interest without need for modification of the intervention itself. Given that this was a scoping review, we judged this was an appropriately inclusive approach to exploring a limited literature, with similar expanded definitions of age categories having been used in previous scoping reviews [43–45]. We did however exclude school-based interventions, (e.g., those which use a school or classroom-based approach in childhood) [46]. This was because our team considered that interventions set within classroom environments would not be replicable in young adults. Where inclusion criteria were borderline, decisions were made on a case-by-case basis and discussed by at least two reviewers (TND and JW).

For the purposes of this review, psychosocial interventions were defined as structured interventions that adopt a psychological, educational, or social framework. This definition was adapted from Ruddy and House, adapting the definition to include digital health interventions, which have taken prominence since the publication of their definition in 2005 [47–49]. In this review, we included search terms pertaining to psychotherapeutic interventions, behavioural interventions, digital interventions, peer/support groups, exercise-based interventions, and psychoeducational interventions.

Study outcomes were checked during the screening process to identify physical health outcomes. We defined physical health broadly as a chronic physical health problem and/or a current behaviour that confers a long-term physical health risk (e.g., unprotected sex, smoking). This definition was formed in collaboration with the Managing Young People with ADHD in Primary Care (MAP) study academic team and research advisory group (RAG) which was composed of people with lived experience [50]. Physical health outcomes included scales (e.g., sleep indices, health-related quality of life), objective measures (e.g., reductions in alcohol consumption, abstinence) and subjective measures (e.g., sleep diaries). Studies were excluded if there was no clear physical health outcome recorded in either the published paper or its supplementary materials. We deliberately did not pre-define specific physical health concerns in our search. This was because, in conjunction with our MAP study advisory team, we felt this would be a more appropriate approach for an initial exploration aimed at capturing the breadth of health problems being addressed. In pre-defining health problems, we considered there would be a risk of excluding studies which incidentally notes physical health outcomes.

Studies from the grey literature were excluded, as well as studies not written in the English language, due to resource limitations. We also excluded non peer-reviewed scientific literature (e.g. dissertations, preprints, conference proceedings). We did not exclude studies where participants received a biological therapy (e.g. medication, bright light therapy), if they also received a psychosocial intervention. This approach enabled a broad spread of relevant included studies and met the review’s objective of identifying feasible interventions.

### Search strategy

The search strategy was developed with information specialists at the at the University of Exeter. Searches were performed using MEDLINE (1946 onwards), Embase (1974 onwards ) and PsycInfo (1803 onwards ), via the Ovid^©^ platform.

**Fig. 1:**
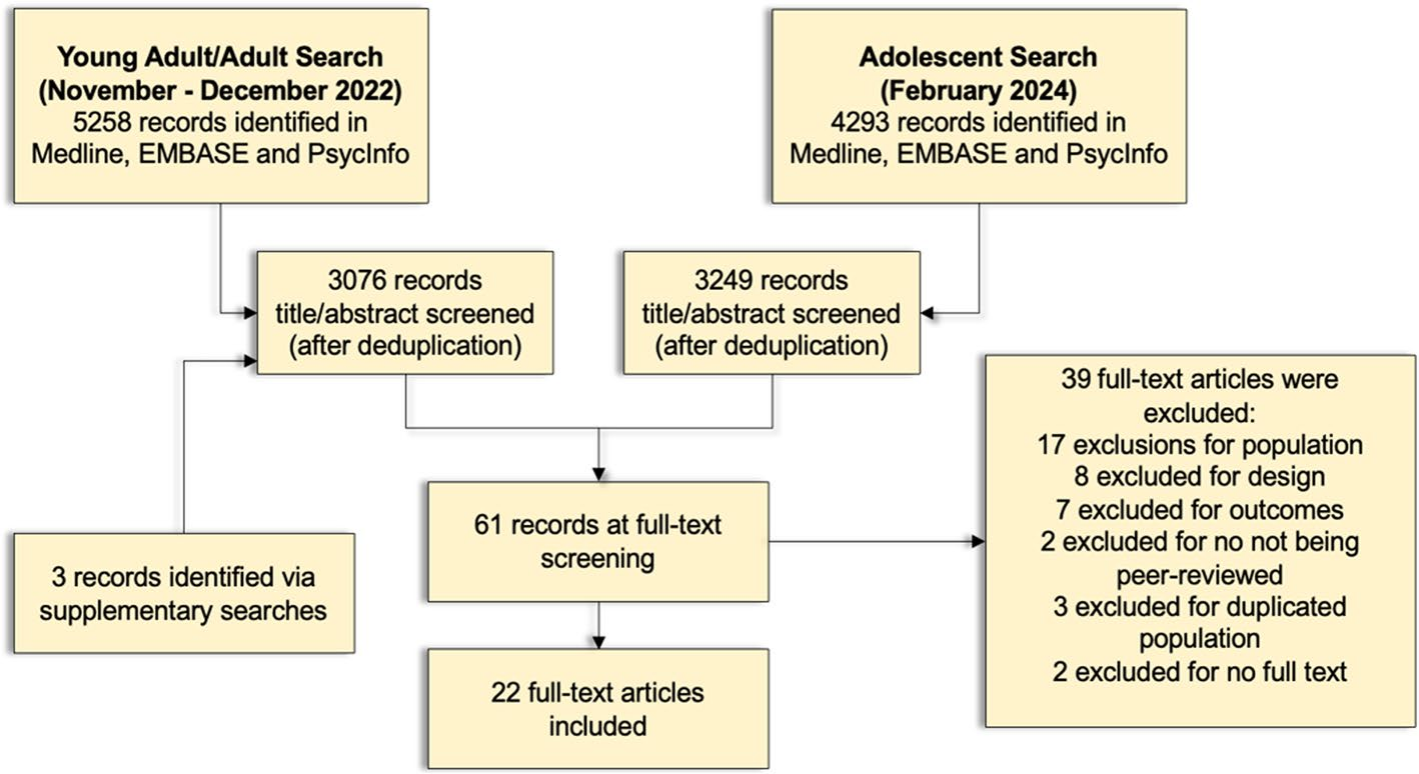
Flow diagram of database searches, title/abstract screening and full text screening

Searching took place in two phases, to ensure adequate inclusion of young people/youths and adults (Fig. 1). In the first phase, we used our young people/youth search terms and our adult terms (search dates 7^th^ October 2022 and 24^th^ November 2022 respectively). The process of search design through the completion of title and abstract screening was from September 2022 to December 2022, with full-text inclusions from this search decided by January 2023.

In order to ensure that we included publications relating to applicable interventions that were tested in a younger population (i.e. 13-17 year olds), a second phase included a search using adolescent search terms, conducted in February 2024, with title/abstract and full text-screening screening completed in March 2024. This search included literature published up until February 2024. Full details of all searches can be seen in supplementary materials.

ADHD search terms were adapted from the search terms used by the National Institute for Care and Health Excellence (NICE) in their development of ADHD guidance [51]. Search terms for psychosocial interventions were adapted from the search terms used by NICE in the development of their guidance for lower back pain and diabetes [52, 53]. These were adapted as appropriate for our search and are available in supplementary materials.

### Study selection

The study selection process is highlighted in Figure 1. The first set of searches (young people/adults, October-November 2022) yielded 5258 results, and the second (adolescents, February 2024) 4293.

These studies were then screened by title and abstract independently by two reviewers (JW, TND), against inclusion criteria, with disagreements resolved by discussion. Where disagreements were not resolved, this was taken to the wider study team and MAP study principal investigator (PI) (AP). Citation chasing was also performed by examining references from relevant review and protocol studies to identify any further studies not found in our search.

This left 61 records for full-text screening. These were again double-screened (JW, TND), with disagreements resolved by discussion, and where necessary the wider study team, and principal investigator (AP), leaving 22 articles which were included at full-text screening. Reasons for full-text exclusions are given in Figure 1.

### Data extraction and charting

Studies were collated using a data extraction form and shared spreadsheet in which the data could be recorded. This data extraction tool was piloted on five papers initially, reviewed and then used for all papers. These were single entered by researchers (JW, AM), with all entries were re-reviewed after first entry (JW). The data extraction tool can be found in supplementary materials.

The data extraction process used narrative synthesis to collate information on what the intervention was targeting (and rationale), what outcomes were measured and how these were measured. This involved extracting study details (i.e., title, author), study design (comparator, methodology), population characteristics (age, gender, location of study), intervention framing of study (i.e. rationale for intervention chosen), ADHD definition (e.g., meeting DSM-V criteria, clinically diagnosed ADHD), relevant additional inclusion/exclusion criteria, primary and secondary outcomes of the studies and measures used, physical health findings, attendance reporting and ADHD symptom findings. Where studies had noted qualitative feedback on the interventions used, this was also collated and charted under outcomes.

Data were then charted in tabular form, by physical health problem addressed, with the extracted details on the studies provided alongside this.

## Results

Twenty-two studies met our inclusion criteria (Table 1). Physical health outcomes targeted or reported included sleep (seven studies), smoking (three studies), substance misuse (four studies), physical activity and/or weight (six studies) and more general/broad physical health outcomes (three studies). It should be noted that one study (Bjork *et al*.) covered two health outcomes (smoking, physical activity) [54]. Studies covered an overall age range of 11-65. Of these, eleven studies examined adolescents, ten studies examined adults (although four did not provide a precise age range), and one study examined both (14–30-year-olds) [55]. Only one included study had participants exclusively between 16-25, but this was a college-based study [56].

**Table 1:** An overview table of the studies, by health issue addressed

Study Title	Location	Health Addressed	Type of Intervention	Study Age Range	Study Method	Sample Size	Study Gender (% Male)	ADHD
Becker et al (2022) [57]	USA	Sleep	Psychoeducation	13-17	Single Group	14	50%	DSM-V Criteria
Van Andel et al (2022) [58]	Netherlands	Sleep	Psychoeducation	18-55	Secondary RCT Data Analysis	49	34.7%	Clinical ADHD (DSM V)
Jernelov et al (2019) [59]	Sweden	Sleep	Behavioural	19-57	Single Group	19	32%	Clinical ADHD
Morgensterns et al (2016) [60]	Sweden	Sleep	Behavioural	19-63	Single Group	98	31.6%	Clinical ADHD
Meyer et al (2022) [55]	Sweden	Sleep	Behavioural	15-18	RCT	184	42.9%	Clinical ADHD
Novik et al (2019) [61]	Norway	Sleep	Behavioural	14-18	RCT	99	NA	Clinical ADHD
Keuppens et al (2023) [62]	Belgium	Sleep	Behavioural	13-17	RCT	92	NA	Clinical ADHD (DSM-V)
Corona et al (2020) [63]	USA	Smoking	Psychoeducation	13-17	RCT	40	72%	Clinical ADHD
Kollins et al (2010) [64]	USA	Smoking	Social	18-50	Two Group (parallel interventions)	46	49%	DSM-IV Criteria
Bjork et al (2020) [54]	Sweden	Multiple	Psychoeducation	Not Given^a^	Single Group	48	40%	Self-reported ADHD
Van Emmerik van Oortmerssen (2019) [65]	Netherlands	Substance Use	Behavioural	18-65	RCT	119	83.2%	DSM-IV Criteria
Meinzer et al (2021) [66]	USA	Substance Use	Behavioural	Not Given ^a^	RCT	113	49.5%	DSM-V Criteria
Riggs et al (2011) [67]	USA	Substance Use	Behavioural	13-18	RCT	303	78.9%	DSM-IV Criteria
Thurstone et al (2010) [68]	USA	Substance Use	Behavioural	13-19	RCT	70	70.9%	Clinical ADHD (DSM-IV Criteria)
Converse et al (2020) [56]	USA	Physical Activity	Exercise	18-23	RCT	21	33%	‘Documented' ADHD
Schoenfelder et al (2017) [69]	USA	Physical Activity	Digital/Exercise	14-18	Single Group	11	46%	Clinical ADHD
Mayer et al (2018) [70]	Germany	Physical Activity	Digital/Exercise	14-30	RCT	NA	NA	DSM-V Criteria
Lindvall et al (2023) [71]	Sweden	Physical Activity	Exercise	Not Given ^a^	RCT	120	NA	Clinical ADHD
Silva et al (2020) [72]	Brazil	Physical Activity	Exercise	11-14	RCT	33	70%	Clinical ADHD
Enggaard et al (2021) [73]	Denmark	Other	Behavioural	13-17	Single Group	10	70%	Clinical ADHD
Mei-Rong et al (2019) [74]	China	Other	Behavioural	Not Given ^a^	Single Group (comparing with data from another study)	124	54%	Clinical ADHD
Geissler et al (2018) [75]	Germany	Other	Behavioural	12-17	RCT	160	NA	Clinical ADHD (DSM-V Criteria)

^a^denotes studies where explicit age ranges are not given, but standard means/deviations or explicit statement indicate that these are studies based in adulthood

In respect to ADHD concept, 14/22 studies were based on clinical diagnosis, whilst 6/22 were criteria based, 1/22 used self-reported ADHD and 1/22 used ‘documented’ ADHD (Table 1). Where studies reported gender (*n=*16), 10 studies were at least 50% female, ranging from a 32% to 83% male sample. 14/22 studies included a physical health outcome as a primary outcome or target, whilst 5/22 studies included physical health as a secondary outcome [55, 56, 60, 61, 74, 75]. 12/22 of the studies were of a psychotherapeutic or behavioural intervention, 4/22 of psychoeducation, 4/22 social/exercise interventions and 2/22 digital interventions. Most of the studies were conducted in the USA (8/22), followed by Sweden (5/22), the Netherlands (2/22) and Germany (2/22), with the others located in Brazil, Norway, Belgium, Denmark and China.

In respect to methodology, 13/22 studies were randomised controlled trials (RCTs) [56, 63, 66–68, 70, 75–77], 6/22 studies were single group (before and after) comparisons [54, 57, 59, 60, 69, 73], one study was secondary analysis of RCT data [58], one study compared a single-group pre/post intervention with a previous study data [74], and finally one study compared two groups before and after comparison (not RCT) [64]. Apart from one study which was unfunded [73], and one study which did not clarify its funding [54], 20/22 studies were funded from non-commercial sources.

### Sleep

Sleep was examined as a health outcome in seven included studies, as described below in Table 2 [55, 57–61, 77]. All used different interventions, which can be broadly divided into psychoeducational interventions and psychotherapeutic interventions (e.g., adapted cognitive or dialectical behavioural therapy). One study examined primarily bright light therapy, however used psychoeducation around sleep in their methodology for both active arms and the placebo arm (hence its inclusion). Only one study included people with diagnosed sleep problems and ADHD, with the others including either a general ADHD population or those with self-reported sleep problems. Four of the studies included are completed or ongoing RCTs, with the rest being pilot/feasibility studies or single group intervention studies. Included studies had small sample sizes (three had fewer than 20 participants, only one had a sample size greater than 100) and covered a wide age range (13-63). Three studies suggested a rationale for their intervention within ADHD in relation to health needs; ADHD symptoms/executive dysfunction impacting on habits and sleep hygiene as the mechanism of sleep problems in ADHD [58, 59, 62] and delayed circadian rhythm/preference [58, 59]. Whilst Becker et al referenced problems of adolescence in sleep, they did not explain the rationale within ADHD [57]. Van Andel *et al* (RCT (melatonin versus placebo versus melatonin and bright light therapy, where all arms received psychoeducation) did not find improvements in sleep for any group [58]. Meyer et al (RCT) also did not find sustained improvements in sleep for either their behavioural or control (psychoeducation group) [55]. However, the single group intervention and pilot studies did find evidence supporting behavioural and psychosocial interventions, including pilot feasibility data from Keuppens *et al* RCT [77]. Results also demonstrated tolerability and feasibility of these sleep interventions in ADHD; all completed studies noted good attendance at and compliance with interventions, whilst Becker *et al.*, Jernelov *et al*. and Keuppens *et al.* noted participants’ satisfaction with interventions. The subjective positive feedback received in Becker *et al.* included increased responsibility for health, working with a therapist and increased knowledge [57]. In Jernelov *et al*., feedback received was the use of routines and structure [59]. For Keuppens *et al.*, thematic analysis generated themes for adolescents around having more control and independence around sleep, and that both parents and adolescents had better understanding of the impact of ADHD on sleep [77].

**Table 2:** Studies examining sleep as a health outcome

Study Title	Type of Study	Population	Intervention Framing	Aims	Intervention	Relevant Outcome Measures	Relevant Findings
Becker et al., 2022 [57]	Pilot/Feasibility study (pre/post)	*N=*14 (50% M), USA 13-17 year olds^a^ without formal sleep disorder diagnoses who met DSM-V ADHD criteria with evidence of sleep problems (as per Pittsburg Sleep Quality Index (PSQI), or less than recommended sleep, or based on Children’s Morning-Eveningness Preference (CMEP) Scale)).	Combination of impairments in ADHD and challenges of adolescence may impact on sleep cycle.	The aim was to evaluate a behavioural intervention in adolescents with evidence of sleep problems.	Six ‘TranS-C’ Sessions—psychoeducational intervention around sleep habits	Subjective sleep quality measures were; Pittsburg Sleep Quality Index (PSQI), Adolescent Sleep-Wake Cycle Scale, (self-reported). Children’s Sleep Habits Questionnaire (CSHQ) (parent-reported). The Pediatric Daytime Sleepiness Scale (PDSS) was also used (parent and self-report). Adolescent Dysfunctional Beliefs about Sleep Scale (DBAS-16).	Significant improvements in PSQI, CSHQ, ASWS and parent-report PDSS scores (*p*<0.001) between pre and post intervention. Significant improvements on self-report PDSS (*p*=0.001). Sleep diaries also demonstrated improvements post intervention. No significant improvements on DBAS. Adolescents rated the intervention quality as ‘high’ or ‘very high’. Positive feedback on interventions included responsibility for health, working with a therapist and increased knowledge.
Van Andel et al., 2022 [58]	Secondary analysis of a randomised control trial (within and between group analyses)	*N=*49 (34.7% M), The Netherlands. 18-55^a^ year olds with clinically diagnosed ADHD and delayed sleep phase syndrome (DSM-V Criteria)	Sleep disorders are common in ADHD, and often related to delayed circadian rhythm.	This paper aimed to study the effects of delayed sleep phase (DSPS) interventions on dim-light melatonin onset (DLMO) in ADHD, in relation to ADHD symptoms.	Melatonin vs Placebo vs Melatonin + BLT All groups received psychoeducation around sleep.	Sleep actometry. Wake after sleep onset duration (WASO). DLMO-midsleep phase angle difference. Circadian rhythm analysis. Sleep diaries. Sleep Hygiene Questionnaire.	No significant improvements on objective or subjective sleep measures over time in any of the groups, despite significant improvements in the Sleep Hygiene Questionnaire.
Jernelov et al., 2019 [59]	Single group interventional study (pre vs post)	*N=*19 (32% M), Sweden. 19-57^a^ year olds with clinically diagnosed ADHD and self-reported sleep problems	Sleep issues are prevalent in ADHD, possibly maintained by executive dysfunction.	The aim was to see if insomnia and ADHD symptoms improve with CBT for insomnia +/- ADHD.	Insomnia and ADHD Cognitive Behavioural Therapy (CBT-i/ADHD)	Insomnia Severity Index (ISI).	Significant improvements in the Insomnia Severity Index (*p*=0.002). Subjective positive feedback on the intervention focussed on routines and structure. Mean session attendance; 7.2/10 sessions.
Morgensterns et al., 2016 [60]	Single group interventional study (pre vs post)	*N=*98 (31.6% M), Sweden. 19-63^a^ year olds with diagnosed ADHD.	ADHD has broad social and daily functioning implications. There is thus far limited evaluation of psychotherapeutic interventions in ADHD.	The aim was to review the acceptability and feasibility of a structured programme for ADHD.	Structured skills training (adapted from dialectical behavioural therapy), 14 2 hour weekly sessions with 45-60m informal conversation afterwards.	Karolinska Sleep Questionnaire. Sheehan Disability Scale (SDS) and Barkley ADHD Functional Impairment subscale.	Improvements in Karolinska Sleep Questionnaire noted as secondary outcome (37.1-33.23 at 3 months post follow-up, p-0.003). Significant improvements in functional impairment scales (*p*<0.001, effect size 0.19 on the Barkley, *p*=.001 and effect size 0.15 on the SDS). 80% of participants attended at least two thirds of sessions.
Meyer et al ., 2022 [55]	Multi-centre randomised Controlled Trial (RCT)	*N=*184 (42.9% M), 15–18-year-olds^a^ with clinically-diagnosed ADHD from outpatient child psychiatry clinics (Sweden)	Psychosocial rather than pharmacological interventions potentially may be more effective in improving daily functioning in ADHD. Given that adolescents typically may struggle with emotional dysregulation and relational problems as well, adapting a structured skills training group (SSTG) from adults into adolescents may be effective.	The aim was to compare the efficacy and acceptability of an SSTG intervention with psychoeducation.	SSTG (*N=*85): 14 weekly 2-hour sessions, blending age-adapted psychoeducation and coping strategies from dialectical behavioural therapy (DBT). Control Psychoeducation (*n=*79): 3x2 hour sessions on ADHD, related problems, lifestyle advice (Sleep and diet). These participants also received a support book on schoolwork.	Karolinska Sleep Questionnaire, Impact of ADHD Symptoms (IAS) (sleep problem component)	Average session attendance was 62% for the intervention group vs 76% for the control group A small between-group difference in sleep problems at T2 (2 weeks after) in favour of the control group. This was not preserved in future follow-ups. There were no other reported significant effects on sleep between or within groups.
Novik et al., 2020 [61]	RCT Protocol	*N=*99 adolescents (14-18^a^ years olds) with diagnosed ADHD, on ADHD medication or medication-resistant and with a Clinical Global Impression Severity score >3 Recruited through two outpatient units in Norway, user organisations, GPs and social media/ newspaper advertisement.	ADHD is highly associated with mental illness and pharmacotherapy may not target this effectively. There is little evidence looking at the broader functional impacts of CBT in ADHD in adolescents.	This study aimed to investigate the efficacy of an ADHD CBT group intervention in adolescents, and to look at functional impairment and psychiatric symptoms.	12 weekly group CBT 90 minute sessions (including a session on sleep), with weekly phone calls and homework, versus treatment as usual (medical treatment with one medical follow-up appointment).	Adolescents’ Sleep Wake Scale	N/A
Keuppens et al., 2023 [62]	RCT protocol	*N=*92 13-17 year olds^a^ with diagnosed ADHD (verified by Kiddie Schedule for Affective Disorder and Schizophrenia Present and Lifetime (K-SADS-PL) DSM-V interview) and sleep problems determined by interview based on DSM-V and International Classification for Sleep Disorders (Third Edition) criteria), stable on ADHD medication without comorbid disorders affecting sleep.	Adolescents with ADHD have poorer and more disrupted sleep than peers, with lack of specific guidance around this. Whilst reasons for this are unclear, sleep hygiene is an important modifiable risk factor for poor sleep. Furthermore, features of ADHD (impulsivity, executive dysfunction) may lead to poorer sleep hygiene. Thus, ADHD and sleep symptoms likely have reciprocal impact.	This study aimed to evaluate a CBT intervention; Sleep Intervention as Symptom Treatment for ADHD (SIESTA) compared to treatment as usual.	CBT-based intervention consisting of seven adolescent sessions and two parent sessions, focussed around a work book, versus treatment as usual (stimulant medication).	Sleep wrist actigraphy, sleep diaries, School Sleep Habits Survey (self/parent report), Chronic Sleep Reduction Questionnaire (parent report), Adolescent Sleep Hygeine Scale.	Pilot evaluation data from 18 participants have been published, with 8 receiving SIESTA. These show satisfaction with SIESTA from parents and adolescents, and subjective improvement in sleep quality, sleep behaviour and daytime sleepiness. Thematic analysis undertaken generated themes around better understanding and control over sleep. Reliable change indices demonstrate significant improvements for all adolescents in at least one Adolescent Sleep Hygiene Scale subdomain [77].

Under population, N denotes sample size, % M denotes the percentage of men in each study

^a^studies which include an age range broader than 16-25

### Smoking

Smoking was examined as a health outcome in three studies, as presented in Table 3 [54, 63, 64]. These studies had an average sample size of 45 and covered both adolescents and adults. One of the studies was a randomised controlled trial (RCT), whilst the other two were a single-group intervention study and an ADHD vs non-ADHD single intervention study, neither with control groups. Two of the studies used psychoeducation, including components about smoking, whilst one of the studies used monetary incentives to encourage participants to stop smoking. In respect to mechanisms, two of the three studies suggested a rationale for their choice of intervention; targeting executive dysfunction in ADHD that may perpetuate smoking [54, 64] and mental health difficulties in ADHD precipitating poor health behaviour [54]. The results were variable. The two studies examining tobacco use (Kollins *et al*., Bjork *et al*.—non-randomised group trials) found no interventions with sustained effects, reporting that participants largely went back to smoking (irrespective of ADHD). Corona *et al.’s* study (also an RCT) found that the attitudes of participants towards substance misuse changed significantly following specific work around tobacco, however they did not examine tobacco use directly. Bjork *et al* and Corona *et al* both noted that participants generally adhered well to the intervention [54, 63]. In respect to specific positives of interventions, Björk *et al.* cite peer support dynamics in their group [54].

**Table 3:** Tables reporting studies on smoking

Study Title	Type of Study	Population	Intervention Framing	Aims	Intervention	Relevant Outcome Measures	Relevant Findings
Corona et al., 2020 [63]	Randomised controlled trial (between and within group comparison)	*N=*40 (72% M), USA. 13-17^a^ year olds who meet DSM-V ADHD criteria.	Adolescents with ADHD are more prone to tobacco use (as well as heavier and earlier use). There is poor evidence around prevention interventions in this context.	This study aimed to assess the feasibility of, and provide preliminary evidence for, a tobacco prevention intervention in adolescents with ADHD.	Supporting Teens Academics Needs Daily Group (STAND-G) with tobacco prevention skills (TPS) from the Strengthening Families Programme	Monitoring the Future Survey (frequency of substance use). Scales examining smoking intention/susceptibility, tobacco refusal intention and beliefs about smoking were studied using scales derived from elsewhere in the literature.	Significant differences in intention to smoke between groups (*p*=0.005, Cohen’s d=0.75). Good adherence to the intervention was noted (94.2%). Mean session attendance across arms was 86%.
Kollins et al., 2010 [64]	Two group intervention study (ADHD vs Non-ADHD)	*N=*46 (49% M), USA. 18-50^a^ year olds who smoke regularly with or without ADHD (ADHD group meeting DSM-IV criteria)	Impulsivity and poorer inhibitory control may explain why those with ADHD find it more difficult to quit smoking. Contingent reinforcement may address this problem.	This study aimed to review the efficacy of monetary incentives in smoking cessation, comparing ADHD and non-ADHD.	Monetary incentives ($4 on day 1 increasing in $4 increments/day abstinent, to a total of $370).	Proportional of sample abstinent from smoking (confirmed using expired carbon monoxide).	Abstinence of 64% at Day 12 in the ADHD group versus 50% in the non ADHD group, and 23% abstinence in the ADHD group 10 days after the intervention finished (vs 9% in non-ADHD group).
Bjork et al., 2020 [54]	Single group intervention study (pre and post intervention)	*N=*48 (mean age 36 (standard deviation, 11)^a^ 40% M), Sweden. Adults with self-reported ADHD and a self-reported mental illness.	Physical comorbidity in ADHD may be related to behavioural and mental health.	The aim was to develop a lifestyle intervention and evaluate its mental/physical health impacts.	20 week health education programme exploring relationships, health education and cognitive support.	Body Mass Index (BMI), waist circumference, Lifestyle-Performance-Health Questionnaire (measuring sedentary habits, eating habits, tobacco use and weekly physical activity), VO2 max (a measure of aerobic fitness).	Slight improvements in weekly physical activity (*p*=0.019), but no significant improvements in tobacco use or eating habits. General health significantly improved (*p*=0.025) as per Lifestyle Performance Health Questionnaire. No significant improvements in Vo2 max. No significant changes in body habitus. At least 70% of sessions were attended by all participants. Peer support highlighted as important to intervention in discussion.

Under population, N denotes sample size, %M denotes the percentage of men in each study

^a^studies which include an age range broader than 16-25

### Substance misuse

Outcomes related to alcohol and substance misuse were examined in four studies, as seen in Table 4 [65–68]. These all had relatively larger sample sizes (range=70-303) and were RCTs. They all included participants who had diagnosable substance misuse disorders, rather than subclinical problematic substance use, (in contrast to the studies of sleep). Two of the studies examined cognitive behavioural therapy (CBT) paradigms, whilst one study evaluated at motivational interviewing (MI) and behavioural action and one used both CBT/MI. Two studies identified a rationale for their choice of intervention; untreated ADHD symptoms being associated with poorer outcomes in substance use disorder [67, 68], and the challenges of people with ADHD within a college environment putting them at greater risk for long-term substance misuse [66]. All four studies (RCTs) reported significant improvements in measured outcomes with a psychotherapeutic intervention, including Riggs *et al.* and Thurstone *et al.* which found that behavioural therapy and medication had comparable effects in the treatment of substance use disorder in patients with ADHD [67, 68].

**Table 4:** Studies detailing alcohol and substance misuse

Study Title	Type of Study	Population	Intervention Framing	Aims	Intervention	Relevant Outcome Measures	Relevant Findings
Van Emmerik van Oortmerssen et al., 2019 [65]	RCT (between and within group comparison)	*N=*119 (83.2% M), The Netherlands. 18-65^a^ year olds with a DSM-IV ADHD and substance use disorder (SUD) other than nicotine.	ADHD has a negative impact on prognosis in SUD, with an unclear efficacy of ADHD medication.	The aim was to evaluate an intervention for SUD in ADHD.	Either CBT for ADHD + SUD or CBT SUD, involving 10-15 sessions.	Days of excessive use in prior week (using time follow back (TFLB) procedures).	Substance misuse decreased across both trial arms, at least at the *p*<0.1 level with moderate effect sizes (CBT SUD 0.44, CBT ADHD SUD 0.45). Attendance means were 12.1/15 for ADHD + SUD CBT group, 8.5/10 for SUD CBT group,
Meinzer at al., 2021 [66]	RCT (between and within group comparison)	*N=*113 (49.5% M), mean age 19.87, (standard deviation 1.44) USA. College students who met DSM-V ADHD criteria, had elevated levels of drinking as per Alcohol Use Disorder Identification Test (AUDIT) scoring	The college environment enables long-term alcohol use problems, in high risk populations (e.g. ADHD). This may be due to alcohol’s immediate rewards (albeit long-term consequences).	The aim was to assess the efficacy of the novel intervention for alcohol misuse in ADHD.	Brief motivating interviewing (BMI) + behavioural action (BA) vs BMI + supportive counselling (SC). Five sessions over 7 weeks.	Brief Young Adult Alcohol Consequences Questionnaire, Daily Drinking Questionnaire, Barkely Functional Impairment Subscale	Reduction in alcohol-related negative consequences, daily drinking and functional impairments for both treatment arms (*p*<0.001).
Riggs et al., 2011 [67]	RCT (between and within group comparison)	*N=*303 (78.9% M), USA. 13-18^a^ year olds with DSM-IV ADHD criteria and one non-tobacco SUD	Untreated ADHD is associated with poorer outcomes in SUD. However, limited research on stimulants in SUD + ADHD exists.	This study aimed to assess the impacts of methylphenidate on SUD.	Medication (modified-release methylphenidate (OROS-MPH)) + CBT (16 week) vs OROS-MPH + CBT	Number of days of substance use in the past 28 days (using TFLB procedures). Mean number of negative urine drug screens.	MPH/CBT and CBT+Placebo groups significant reduction, no between group difference (*p*<0.0001). No difference in medication abuse/diversion between groups. Significantly more negative urine drug screens in OROS-MPH+CBT group (*p*=0.05). Session attendance not statistically significantly different between arms (numbers not given).
Thurstone et al., 2010 [68]	RCT	*N=*70 (79% M, mean age 16.1) 13-19^a^ year olds with diagnosed ADHD (DSM-IV, and DSM-IV checklist score ≥22) and a DSM-IV non-nicotine substance use disorder	SUDs are common in ADHD, and ADHD is associated with poorer SUD outcomes. There is limited evidence surrounding ADHD treatment on SUDs in adolescents.	This study hypothesised that atomoxetine + MI/CBT would be more effective than placebo + MI/CBT.	Atomoxetine (titrated to participant) or placebo alongside 12 one hour sessions of MI/CBT for SUD, with up to three family sessions. This was over twelve weeks.	Self-reported number of days of substance use (excluding nicotine) over past 28 days. Baseline and fortnightly urinary drug screens.	Both groups demonstrated significant reduction from baseline in number of days of substance use (mean decrease 4.01 days, *p*=0.0015), but did not differ from each other. Groups did not differ on number of negative urinary drug screens (*p*=0.972).

Under population, N denotes sample size, %M denotes the percentage of men in each study

^a^studies which include an age range broader than 16-25

### Physical activity/weight

Studies reporting physical activity or weight outcomes were much more heterogenous in their design, including two RCT protocols, two completed RCTs and two single group interventions (Table 5). Studies in this category had generally small sample sizes (n<50), except Lindvall et al (*N=*120) [71] and comprised a younger adult demographic (11-30). Only two studies provided a rationale for intervention explicitly highlighting health in ADHD (both referencing poor health behaviour in ADHD) [54, 71]. All involved promoting physical activity, through structured exercise classes, wearable technology/social media and psychoeducation respectively. Furthermore, Schoenfelder *et al.* report qualitative feedback that the intervention increased awareness of activity levels and ADHD symptoms [69]. Both RCTs (Silva et al, Converse et al [56, 72]) reported improved physical functioning (Converse et al using a questionnaire, Silva et al using objective biometrics), as did both single group intervention studies (Schoenfelder et al finding an increase in step count, Bjork et al in weekly physical activity [54, 69]).

**Table 5:** Studies addressing physical activity interventions

Study Title	Type of Study	Population	Intervention Framing	Aims	Intervention	Relevant Outcome Measures	Relevant Findings
Converse et al., 2020 [56]	RCT (between group comparisons)	*N=*21 (33% M), USA. 18-23 year olds undergraduates with a documented ADHD diagnosis.	Factors such as medication intolerance or risk of diversion mean non-pharmacological options are required for ADHD treatment.	The aims here were to study feasibility of maintaining a 7-week Tai Chi intervention.	Tai Chi sessions (2x1hrs/week) versus ‘box blast’ fitness class vs inactive trial	PSQI, SF-36 (physical functioning).	Significant improvements in physical functioning (SF-36) (*p*<0.0001), non-significant improvements in sleep as per PSQI. Mean attendance at tai chi 3.7/14 classes (not recorded for active control). Subjective feedback
Schoenfelder et al., 2017 [69]	Single group intervention study (pre vs post comparison)	*N=*11 (46% M), USA. 14-18^a^ year olds with clinically-diagnosed ADHD	Low acceptability of pharmacological ADHD treatment in adolescents leaves them at risk of behavioural and functional impairment. Physical activity may help ADHD symptoms and mood.	The study aimed to see the feasibility and acceptability of an m-health intervention to increase daily physical activity amongst adolescents with ADHD.	Four weeks of wearing a FitBit and receiving individualised activity goal, with a Facebook group and SMS messages	Step count.	Significant increase in steps (*p*=0.005). Themes from qualitative interviews included increased awareness of activity levels and ADHD symptoms. Suggested improvement was reminders within the app.
Bjork et al., 2020 [54]	Single group intervention study (pre vs post comparison)	*N=*48 (mean age 36 (standard deviation, 11)^a^ 40% M), Sweden. Adults with self-reported ADHD and a self-reported mental illness.	Physical comorbidity in ADHD may be related to behavioural and mental health.	The aim was to develop a lifestyle intervention and evaluate its mental/physical health impacts.	20 week health education programme exploring relationships, health education and cognitive support.	Body Mass Index (BMI), waist circumference, Lifestyle-Performance-Health Questionnaire (measuring sedentary habits, eating habits, tobacco use and weekly physical activity), VO2 max (a measure of aerobic fitness).	Slight improvements in weekly physical activity (*p*=0.019) and more physical activity, but no significant improvements in tobacco use or eating habits. General health significantly improved (*p*=0.025) as per Lifestyle Performance Health Questionnaire. No significant improvements in Vo2 max. No significant changes in bdoy habitus. At least 70% of sessions were attended by all participants.
Mayer et al., 2018 [70]	RCT (between /within group comparisons)	14-30 year olds, meeting DSM-V ADHD criteria for ADHD. Protocol Study, Germany	There is a significant association between ADHD and obesity. There is poor evidence around treatment.	The aim is to understand the efficacy of an exercise/bright light therapy intervention on depression and obesity.	Bright light therapy (6x/week, 30m exposure) and an app-facilitated exercise intervention around aerobic/muscle strength training.	Physical Activity Readiness Scale, questions on sleep behaviour, EuroQol-5 Dimensions-3 Levels (EQ-5D-3L; a measure of self-reported health), SF-36 (self-reported health), Munich Chronotype Questionnaire, lower limb explosive strength, grip strength, BMI, body composition, waist circumference, International Fitness Scale, concentrations of melatonin, cortisol, leptin, and ghrelin, Fagerström Test for Nicotine Dependence, Yale Food Addiction Scale.	Not recorded.
Lindvall et al., 2023 [71]	RCT Protocol	*N=*120 adults with diagnosed ADHD from an outpatient psychiatry clinic (Sweden) and internet/ social-media channels	Exercise has positive effects on ADHD symptoms. There is evidence that people with ADHD struggle with routines and being active. It is possible that a structured physical activity intervention may be beneficial.	This study aims to evaluate a 12 week mixed exercise programme (Stöd i Aktivitet, Rörelse och Träning (START) intervention), with or without cognitive skills training, in relation to ADHD symptoms, as well as physical/cognitive functions and experiences of daily living.	12 week physiotherapist-led physical training (START, 45 minute sessions, 3 per week) (*N=*40), +/- occupational therapist led person-centred intervention (6 sessions) (*N=*40), versus treatment as usual (i.e. continued normal pharmacological/non-pharmacological management) (*N=*40)	EQ-5D-5 L (EuroQol measure of health status and quality of life), Ekblom-Bak submaximal exercise test (VO2 max), Flamingo balance test, grip strength, Body Awareness Scale-Movement Quality and Experience	Prospective (estimated completion of trial in Autumn 2024)
Silva et al (2020) [72]	RCT (within group comparison)	*N=*33 children (aged 11-14) with previously diagnosed ADHD. Brazil.	Individuals with ADHD may have poorer executive function and higher levels of mood disorders/stress than peers. Medication may cause adverse side effects. Exercise has eben noted to be effective in modulated both mood and ADHD symptoms.	This study aimed to evaluate a swimming training intervention on physical/mental health outcomes.	Swimming training, (45 minute sessions, 2/week, 8 weeks). Control arm intervention ‘untrained’ (no further detail given).	Motor coordination tests and laterality of lower limbs, flexibility (sit and reach test), abdominal resistance test	Per protocol analysis shows significant improvements in motor coordination and laterality of lower limbs, flexibility and abdominal resistance measures in the trained group (*p*<0.05), but not in the untrained group. 14/18 had >70% intervention adherence in the trained group.

Under population, N denotes sample size, %M denotes the percentage of men in each study

^a^studies which include an age range broader than 16-25

### Unspecified physical health outcomes

Three studies examined unspecified physical health outcomes related to quality of life and did not fit well into other categories (Table 6) [73–75]. Enggaard *et al.* reported a study of adolescents with a comorbid physical health disorder, examining guided self-determination as a way of improving their engagement in physical healthcare, given the association of ADHD with physical comorbidity. They found that guided self-determination was effective in improving patients’ self-confidence in managing their conditions, and that adolescents were positively engaged in creating the self-management strategies. The second study examined effects of medication versus cognitive behavioural therapy in 124 young adults on core ADHD symptoms and secondarily recorded improvements in physical health as part of questions on the World Health Organisation (WHO) quality of life scale [78]. This study [74] did not identify a rationale for their intervention’s impact physical health. Thirdly, Geissler et al developed a modular treatment programme for adolescents with continual ADHD-related impairment (under routine care). Their rationale was the breadth of functional impairment faced by adolescents with ADHD, and the lack of related interventions. Their developed RCT protocol includes a health-related quality of life questionnaire as a secondary outcome, and their intervention includes a module on substance use [75].

**Table 6:** Studies addressing a component of physical health without a singular defined physical health problem

Study Title	Type of Study	Population	Intervention Framing	Aims	Intervention	Relevant Outcome Measures	Relevant Findings
Enggaard et al., 2021 [73]	Single group intervention study (pre vs post comparison)	*N=*10 (70% M), Denmark. 13-17^a^ year olds with clinically-diagnosed ADHD and a comorbid medical disorder.	Children with ADHD are at highest risk of medical comorbidity. Furthermore, ADHD is associated with more difficulties in self-management and greater dependence on parents.	The aim was to study the impact of intervention on the support from nurses and parents, and the adolescent’s self-management .	Guided self-determination around professional communication and self-reflection.	Patient Activation Measure (PAM), Healthcare Climate Questionnaire (HCCQ), Perception of Parents Scale (POPS)	Slight increases in PAM (statistical significance not provided), stability of HCCQ and POPS. Qualitatively, improvements in self-confidence and strategy development. 7/10 adolescents attended all sessions.
Mei-Rong et al., 2019 [74]	Single group intervention study (pre vs post comparison) compared with previous study data	*N=*124 (54% M), China (Mean age 27.1±4.81/26.8±5.4^a^ (CBT vs CBT + medication groups). Sample was drug naïve outpatients with adult ADHD diagnoses	ADHD medications have problems with adherence, side-effects and addiction. Psychotherapy has demonstrable neurobiological impacts that may help to remedy the impacts of ADHD on neuronal networks.	This study aimed to review the effects of medication and psychotherapy on ADHD symptoms and executive functions.	Medication (methylphenidate or atomoxetine) versus medication + CBT for 12 weeks.	World Health Organization Quality of Life-Brief version (WHOQOL- BREF)	Significant improvements in the WHOQOL-PREF in the CBT only group, but not in the CBT + medication group (*p<*0.01, Cohen’s D=0.01).
Geissler et al., 2018 [75]	RCT Protocol	*N=*160 12-17^a^ year olds with diagnosed ADHD (DSM-V criteria) with continued marked impairment despite at least 6 months of routine ADHD care (by Clinical Global Impression (CGI) severity scale)	ADHD is associated with a range of poor health, functional and social outcomes. Targeted psychosocial interventions in adolescents are required to improve function and impairment.	This study aims to assess a novel intervention (Individualised Modular Treatment Programme (IMTP)), versus active control (telephone assisted self-help (TASH) for parents).	Following four weeks of treatment as usual, participants still with marked impairment will be randomised to IMTP or TASH. IMTP consists of 10 sessions (1 hour long, over 12 weeks) of a CBT intervention. It consists of 4 mandatory and 6 tailored sessions related to ADHD. It includes a session on substance use. TASH consists of 8 leaflets on parenting adolescents with ADHD, accompanied by 10 sessions of counselling via phone (30 minutes each).	Health-Related Quality of Life Questionnaire for Children and Young People (KIDSCREEN-10)	

Under population, N denotes sample size, %M denotes the percentage of men in each study

^a^studies which include an age range broader than 16-25

## Discussion

This scoping review aimed to identify psychosocial interventions that have been designed for physical health problems in ADHD, and which physical health problems they target. We found 22 studies of interventions which measured at least one physical health outcome, with 16 specifically targeting physical health outcomes. In the other studies, measures of physical health (including sleep quality or health-related quality of life measures) were included as secondary outcomes of interventions primarily targeting reduction of core ADHD symptoms [55, 56, 60, 61, 74, 75]. Included studies were grouped under five categories, dependent on the outcomes explored. These were sleep, smoking, substance misuse, physical activity/weight, and general health outcomes, utilising psychoeducational, behavioural and social paradigms (Table 1).

The main finding from this scoping review is a relative paucity of research into interventions targeting physical health outcomes in ADHD, and furthermore the lack of larger programmes of research aiming to address the health problems identified. Generally, the included studies lacked detail on the framing and theoretical basis both of individual health problems (who is affected, and how that health problem is quantified) as well as of health problems in ADHD in general (the 'mechanism' of ill health targeted by such interventions). Fortunately, there is some similarity amongst the identified literature explored in respect to the psychosocial interventions used and identified positive aspects of interventions, which may form a basis for the development of a more coherent evidence base in this field.

### Framing of health problems

There is substantial heterogeneity in this literature in respect to how health problems were defined and measured, with variable inclusion criteria and outcome measures between studies. For example, when exploring sleep, some studies examined those with formal sleep diagnoses [58], whilst others specifically excluded those with diagnosed sleep problems [59, 62]. In relation to outcome measures, smoking was conceptualised in a different way by each of the studies included (attitudes to tobacco, carbon monoxide levels and self-reported reductions). Some studies used standardised quality of life measures, such as the WHOQL-BREF, SF-36, and KIDSCREEN-10 [56, 74, 75]. However, quality of life measures are of limited utility in assessing physical health outcomes, often being too broad and multi-factorial. We also note a recent scoping review finding that the SF-36 is frequently erroneously reported as a global measure of quality of life, which although Converse and colleagues did not do, highlights a wider problem with the misapplication of quality-of-life measures [79]. The heterogeneity of included studies’ outcome measures highlights the need for consensus in respect to measures used in assessing the physical health outcomes of populations with ADHD. It was interesting that three of the six studies that did not explicitly target a health problem used sleep outcome measures in behavioural interventions [55, 60, 61], which may highlight the importance of sleep to young people and families, and the impact poor sleep has on symptoms and functioning [80, 81].

Such extensive variability in inclusion criteria and outcome measurements limits both the clinical and academic applicability of studies’ findings. This variability is likely to result from the absence of a common framework that mechanistically relates ADHD and physical health outcomes.

Whilst there was some commonality in intervention modalities (e.g., behavioural interventions, educational interventions), authors tended not to explain clearly which mechanism within ADHD their health intervention was targeting, beyond a select few [54, 57–59, 62, 64, 66, 71, 73]. Challenges surrounding poor conceptual framework of mechanisms of ADHD in relation to health are alluded to in the discussions of some of the included studies [54, 58, 59, 64, 66, 67, 73]. As mentioned in the introduction, the ‘causal pathway’ of increased health risk in ADHD is likely to be complex and multifactorial, however a sound understanding and explicit logic model is an important basis for the development of preventative interventions in this population. Findings from this scoping review suggest that that clearer framing of the problem is required to properly develop interventions, through better definition of health problems with inputs from existing research and stakeholder perspectives (which may explain the currently disjointed view of this field) [82].

### Positive aspects of interventions

Psychoeducation was common amongst health interventions studied, with 4/22 solely examining a psychoeducational intervention, and Meyer et al comparing a behavioural intervention with psychoeducation as control [55]. These studies tended to recruit younger participants who were ‘at risk of’ particular health problems, with only Bjork *et al* using psychoeducation in an adult context. This raises questions about where future research work should focus, primary or secondary prevention in young people and adults with ADHD.

‘Self-efficacy’ or independence over one’s health was also a concept referenced explicitly in the qualitative feedback from participants included in several of the studies we reviewed [57, 73, 77]. Furthermore, all the interventions in included studies all required commitment to interventions and required people to actively engage in their own care, the importance of which has been studied previously in patients with chronic conditions [83–85]. Self-efficacy is widely cited as being important in ADHD management [86, 87]. This is explicitly highlighted by Enggaard *et al.*, who demonstrated that their guided self-determination intervention promoted efficacy and strategy formation amongst patients with ADHD [73].

Regular and consistent interventions (regular sessions, commitment to a regimen), were explicitly highlighted in the qualitative participant feedback on several interventions [59, 69]. This is particularly pertinent in ADHD, where difficulty with day-to-day structure and organisation is something that people highlight as a contributor to health and social outcome inequalities [88–90].

Peer dynamics were also referenced by several papers [54, 56]. Bjork *et al.* reported that participants found the peer support dynamic of such interventions useful, whilst Converse *et al.* reported that participants from an earlier survey used in the development of their intervention would have preferred a mixed ADHD/non-ADHD group [91]. From a brief review of the literature, the perspectives on peer support in adult ADHD have not yet been formally studied but could be looked at in future work. It should be noted that, in discussions about ADHD in online spaces, community and identity appear to be important themes in living with ADHD [87, 92].

If the literature in this field were more coherent, it would make it easier to explore facets of interventions in this field more rigorously, using methods such as intervention component analysis. This would be especially interesting given the findings of Meyer et al, which suggest comparable effects between psychoeducation and behavioural intervention [55].

## Strengths and limitations

This study addresses a novel research question in the literature and our search strategy identified papers in line with research aims. By not defining physical health in our search strategy, we were able to identify a broad range of interventions, targeting for example sleep, smoking, alcohol/substance misuse, physical activity, weight, and physical comorbidity.

Limitations of our scoping review include challenges around defining population age range. Of the 22 studies included, only one study explicitly fell within the 16-25 age range [56]. Whilst it may have been preferable to strictly apply the lower age limit of 16 years, doing so would have risked losing studies with applicability to our 16-25 age group (e.g., Schoenfelder and colleague’s study of digital health, many of the sleep behavioural interventions) [57, 61, 69]. The same would apply to the upper age limit, where studies such as Björk et al would be excluded if a strict age basis was applied (despite this study having clear relevance to our question) [54]. Therefore, we adopted a pragmatic approach, informed by consultation with MAP study colleagues and our RAG. We accept that the interventions would likely have different effects than those reported by the studies, if they were to be repeated in a strictly 16-25 age group. This would be an important subject of future work, supported by framework development.

By defining ‘types’ of interventions we were interested in for our search (psychotherapeutic, behavioural, technological, support groups, exercise-based, psychoeducational) we may have inadvertently precluded the inclusion of other interventions in the field. However, to deliver the review with the resources available, and following consultation with our RAG, it was decided to prioritise an open approach to defining physical health problems, which came at the cost of being more restrictive in terms of types of intervention reviewed. As this research area matures, and concepts related to ADHD and physical health become more clearly defined, it will become easier to conduct evidence syntheses of literature on this topic.

Furthermore, it was notable that there were limited interventions surrounding established chronic physical disorders targeted at adults with ADHD, given the known associations of ADHD with chronic health problems [12, 14, 93]. However, this is likely because our search filters examining psychosocial interventions would not have been inclusive of tailored medical interventions (e.g. if a study were examining supporting people with ADHD and diabetes in their medication compliance). A focussed review of chronic disease management in ADHD in adults would be useful in exploring this important area.

## Conclusion

This scoping review set out to identify existing psychosocial interventions for physical health in ADHD, with a focus on interventions applicable to a transition care age range (16-25 year olds). Findings demonstrate that whilst such interventions have been developed and reported, the small evidence base surrounding them limits their current application. Future work in this field needs to focus on the development of a conceptual framework for the origins of the physical health challenges and linked health inequalities we see in ADHD. Alongside this, more research is needed into creating standardising how health outcomes are measured and reported in ADHD research in this field, such that evidence can be better synthesised and ultimately realised into clinical applications.

### Supplementary Information

Supplementary Material 1

Supplementary Material 2

Supplementary Material 3

## Data Availability

The datasets generated and/or analysed during the current study are available via the MEDLINE (https://www.nlm.nih.gov/medline/medline_overview.html), APA PsycInfo (https://www.apa.org/pubs/databases/psycinfo) and EMBASE (https://www.embase.com/) databases.

## Abbreviations

ADHD: Attention deficit hyperactivity disorder
CBT: Cognitive Behavioural Therapy
DSM-V: Diagnostic and Statistical Manual of Mental Disorders Fifth Edition
K-SADS-PL: Kiddie Schedule for Affective Disorders and Schizophrenia Present and Lifetime
MAP: Mapping ADHD services in Primary Care Study
NICE: National Institute of Health and Care Excellence
RAG: Research Advisory Group
RCT: Randomised Control Trial
SF-36: 36-item Short Form Survey
WHO: World Health Organisation
WHOQL-BREF: World Health Organisation Quality of Life (Brief) Scale
