# Supplementary material for: Psychosocial interventions for improving the physical health of young people and adults with attention deficit hyperactivity disorder: a scoping review
Source: BMC Psychiatry. 2024 Aug 20;24:569. doi: 10.1186/s12888-024-06009-2 (PMC11337789; doi:10.1186/s12888-024-06009-2)
Supplement: Supplementary file 1 — Supplementary Material 1 [file 12888_2024_6009_MOESM1_ESM.docx]

| **Variable** | **Response** |
| --- | --- |
| Study Title |  |
| Year of publication |  |
| Study ID (First author et al, Year) |  |
| Study design |  |
| What were the premises/aims of the study? |  |
| What is the identified health risk within the study? |  |
| What is the age range of the study population? |  |
| What is the sample size of the study (please give the gender split as a % of men e.g. n=50 (50% M)) |  |
| Where is the study based? |  |
| What were the inclusion criteria for the study? |  |
| What were the exclusion criteria for the study? |  |
| What is the proposed intervention? |  |
| What are the primary outcomes of this study (please put an after the relevant physical health indicators)? |  |
| What were the secondary outcomes (if any) of this study? |  |
| In regards to the physical health outcome measure, what do the results demonstrate? |  |
| Are there limitations noted by the study noted by the authors? |  |
