# Supplementary material for: Psychosocial interventions for improving the physical health of young people and adults with attention deficit hyperactivity disorder: a scoping review
Source: BMC Psychiatry. 2024 Aug 20;24:569. doi: 10.1186/s12888-024-06009-2 (PMC11337789; doi:10.1186/s12888-024-06009-2)
Supplement: Supplementary file 3 — Supplementary Material 3 [file 12888_2024_6009_MOESM3_ESM.docx]

**Young Adult Search: Medline (1946-present)/Embase (1974-present)**

| 1 | "Attention Deficit and Disruptive Behavior Disorders"/ or Attention Deficit Disorder with Hyperactivity/ |
| --- | --- |
| 2 | ((attenti* or disrupt*) adj3 (adolescent* or adult* or behav* or child* or class or classes or classroom* or condition* or difficult* or disorder* or learn* or people or person* or poor or problem* or process* or youngster*)).ti. |
| 3 | ((attenti* or disrupt*) adj3 disorder*).ab. |
| 4 | (adhd or addh or ad hd or ad??hd).ti,ab. |
| 5 | (attenti* adj3 deficit*).ti,ab. |
| 6 | (((hyperkin* or hyper kin*) adj1 (syndrome* or disorder*)) or hkd).ti,ab. |
| 7 | (minimal brain adj2 (dysfunct* or disorder*)).ti,ab. |
| 8 | 1 or 2 or 3 or 4 or 5 or 6 or 7 |
| 9 | Mobile Applications/ |
| 10 | exp Internet/ |
| 11 | exp Cell Phone/ |
| 12 | exp Computers, Handheld/ |
| 13 | Medical Informatics Applications/ |
| 14 | Therapy, Computer-Assisted/ |
| 15 | (app or apps).ti,ab. |
| 16 | (online or web or internet or digital*).ti. |
| 17 | ((online or web or internet or digital*) adj3 (based or application* or intervention* or program* or therap*)).ab. |
| 18 | (phone* or telephone* or smartphone* or cellphone* or smartwatch*).ti. |
| 19 | ((phone* or telephone* or smartphone* or cellphone* or smartwatch*) adj3 (based or application* or intervention* or program* or therap*)).ab. |
| 20 | (mobile health or mhealth or m-health or ehealth or e-health or emental or e-mental).ti. |
| 21 | ((mobile health or mhealth or m-health or ehealth or e-health or emental or e-mental) adj3 (based or application* or intervention* or program* or therap*)).ab. |
| 22 | (mobile* adj3 (based or application* or intervention* or device* or technolog*)).ti,ab. |
| 23 | or/9-22 |
| 24 | exp Behavior Therapy/ |
| 25 | exp *cognitive therapy/ |
| 26 | *mindfulness/ |
| 27 | (therap* or psychotherap*).ti,ab. |
| 28 | (cbt or mindful*).ti,ab. |
| 29 | ((cognitive or cognition or behavio?r*) adj2 (therap* or psychotherap*)).ti,ab. |
| 30 | (psych* adj2 (therap* or treatment*)).ti,ab. |
| 31 | "psychoeducation".ti,ab. |
| 32 | adaptation, psychological/ or feedback, psychological/ |
| 33 | feedback, psychological/ or psychosocial intervention/ or psychotherapy, brief/ |
| 34 | or/24-33 |
| 35 | exp exercise/ |
| 36 | exp exercise therapy/ |
| 37 | exp "physical education and training"/ |
| 38 | (pilates or yoga or mckenzie or feldenkrais or swim* or walk* or run* or jog* or treadmill* or tread mill*).ti,ab. |
| 39 | (aerobic* adj (exercise* or train* or therap*)).ti,ab. |
| 40 | ((corrective* or biomechanic*) adj (exercise* or train* or therap*)).ti,ab. |
| 41 | (stretch* adj3 (active* or passive* or relax* or static* or dynamic* or gentl* or ballistic* or force* or isometric or technique* or exercis* or therap*)).ti,ab. |
| 42 | ((strength* or stabil* or program* or train* or therap* or technique* or treat*) adj3 exercise*).ti,ab. |
| 43 | (fitness* adj3 (prog! ram* or t! rain* or therap*)).ti,ab. |
| 44 | (tai ji or tai chi or taichi or taiji or taijiquan).ti,ab. |
| 45 | (qigong or ch'i k#ng or ch'i g#ng or chi k#ng or chi g#ng or qi k#ng or qi g#ng).ti,ab. |
| 46 | yoga/ |
| 47 | exercise movement techniques.mp. [mp=title, book title, abstract, original title, name of substance word, subject heading word, floating sub-heading word, keyword heading word, organism supplementary concept word, protocol supplementary concept word, rare disease supplementary concept word, unique identifier, synonyms, population supplementary concept word, anatomy supplementary concept word] |
| 48 | Life Style/ |
| 49 | or/35-48 |
| 50 | Self Help/ |
| 51 | Group Support/ |
| 52 | support* group*.tw. |
| 53 | group* support*.tw. |
| 54 | peer* support*.tw. |
| 55 | self help group*.tw. |
| 56 | (therapeutic adj social club*).tw. |
| 57 | ((nonmedical or non professional* or lay or layperson* or peer* or voluntary or patient) adj2 (instructor* or tutor* or educator* or consultant* or leader* or expert* or advisor* or facilitat* or deliver* or mento! r* or led! or guide* or aide* or run)).tw. |
| 58 | or/50-57 |
| 59 | 23 or 34 or 49 or 58 |
| 60 | (young adj3 (person or people)).ti,ab. |
| 61 | Young Adult/ |
| 62 | youth.ti,ab. |
| 63 | or/60-62 |
| 64 | 8 and 59 and 63 |
| 65 | (assign* or allocat* or volunteer* or placebo*).ti,ab. |
| 66 | exp controlled clinical trials as topic/ or feasibility studies/ or pilot projects/ |
| 67 | controlled clinical trial.pt. |
| 68 | randomi#ed.ab. |
| 69 | trial.ab. |
| 70 | groups.ab. |
| 71 | or/65-70 |
| 72 | 64 and 71 |

**PsycInfo (1806-present) Young Adult**

| # | Search History |
| --- | --- |
| 1 | Attention Deficit Disorder with Hyperactivity/ |
| 2 | ((attenti* or disrupt*) adj3 (adolescent* or adult* or behav* or child* or class or classes or classroom* or condition* or difficult* or disorder* or learn* or people or person* or poor or problem* or process* or youngster*)).ti. |
| 3 | ((attenti* or disrupt*) adj3 disorder*).ab. |
| 4 | (adhd or addh or ad hd or ad??hd).ti,ab. |
| 5 | (attenti* adj3 deficit*).ti,ab. |
| 6 | (((hyperkin* or hyper kin*) adj1 (syndrome* or disorder*)) or hkd).ti,ab. |
| 7 | (minimal brain adj2 (dysfunct* or disorder*)).ti,ab. |
| 8 | or/1-7 |
| 9 | Mobile Applications/ |
| 10 | exp Internet/ |
| 11 | exp Mobile Phones/ |
| 12 | exp Computers/ |
| 13 | exp Digital Interventions/ |
| 14 | (app or apps).ti,ab. |
| 15 | (online or web or internet or digital*).ti. |
| 16 | ((online or web or internet or digital*) adj3 (based or application* or intervention* or program* or therap*)).ab. |
| 17 | (phone* or telephone* or smartphone* or cellphone* or smartwatch*).ti. |
| 18 | ((phone* or telephone* or smartphone* or cellphone* or smartwatch*) adj3 (based or application* or intervention* or program* or therap*)).ab. |
| 19 | (mobile health or mhealth or m-health or ehealth or e-health or emental or e-mental).ti. |
| 20 | ((mobile health or mhealth or m-health or ehealth or e-health or emental or e-mental) adj3 (based or application* or intervention* or program* or therap*)).ab. |
| 21 | (mobile* adj3 (based or application* or intervention* or device* or technolog*)).ti,ab. |
| 22 | or/9-21 |
| 23 | exp Behavior Therapy/ |
| 24 | exp Cognitive Behavior Therapy/ |
| 25 | exp Dialectical Behavior Therapy/ |
| 26 | exp Biopsychosocial Approach/ |
| 27 | exp Mindfulness/ |
| 28 | exp Meditation/ |
| 29 | exp Mindfulness-based interventions/ |
| 30 | (therap* or psychotherap*).ti,ab. |
| 31 | (cbt or mindful*).ti,ab. |
| 32 | ((cognitive or cognition or behavio?r*) adj2 (therap* or psychotherap*)).ti,ab. |
| 33 | (psych* adj2 (therap* or treatment*)).ti,ab. |
| 34 | exp Psychoeducation/ |
| 35 | exp Clinical Psychology/ |
| 36 | exp Biopsychosocial Approach/ |
| 37 | exp Psychosocial Rehabilitation/ |
| 38 | exp Interdisciplinary Treatment Approach/ |
| 39 | or/23-38 |
| 40 | exp exercise/ |
| 41 | exp Physical Education/ |
| 42 | (pilates or yoga or mckenzie or feldenkrais or swim* or walk* or run* or jog* or treadmill* or tread mill*).ti,ab. |
| 43 | (aerobic* adj (exercise* or train* or therap*)).ti,ab. |
| 44 | ((corrective* or biomechanic*) adj (exercise* or train* or therap*)).ti,ab. |
| 45 | (stretch* adj3 (active* or passive* or relax* or static* or dynamic* or gentl* or ballistic* or force* or isometric or technique* or exercis* or therap*)).ti,ab. |
| 46 | ((strength* or stabil* or program* or train* or therap* or technique* or treat*) adj3 exercise*).ti,ab. |
| 47 | (fitness* adj3 (program* or train* or therap*)).ti,ab. |
| 48 | (tai ji or tai chi or taichi or taiji or taijiquan).ti,ab. |
| 49 | (qigong or ch'i k#ng or ch'i g#ng or chi k#ng or chi g#ng or qi k#ng or qi g#ng).ti,ab. |
| 50 | yoga/ |
| 51 | exercise movement techniques.mp. [mp=title, abstract, heading word, table of contents, key concepts, original title, tests & measures, mesh word] |
| 52 | Lifestyle/ |
| 53 | exp Lifestyle Changes/ |
| 54 | or/40-53 |
| 55 | Support Groups/ |
| 56 | Social Group Work/ |
| 57 | peer* support*.tw. |
| 58 | Peer Counseling/ |
| 59 | self help group*.tw. |
| 60 | (therapeutic adj social club*).tw. |
| 61 | ((non medical or non professional* or lay or layperson* or peer* or voluntary or patient) adj2 (instructor* or tutor* or educator* or consultant* or leader* or expert* or advisor* or facilitat* or ! deliver* ! or mentor* or led or guide* or aide* or run)).tw. |
| 62 | or/55-61 |
| 63 | 22 or 39 or 54 or 62 |
| 64 | (young adj3 (person or people)).ti,ab. |
| 65 | Emerging Adulthood/ |
| 66 | youth.ti,ab. |
| 67 | or/64-66 |
| 68 | 8 and 63 and 67 |
| 69 | (assign* or allocat* or volunteer* or placebo*).ti,ab. |
| 70 | exp Clinical Trials/ |
| 71 | Randomised Clinical Trials/ |
| 72 | Treatment Effectiveness Evaluation/ |
| 73 | (pilot or feasibility).ti,ab. |
| 74 | Followup Studies/ |
| 75 | randomi#ed.ab. |
| 76 | trial.ab. |
| 77 | groups.ab. |
| 78 | or/69-77 |
| 79 | 68 and 78 |

**Medline (1946-present)/Embase (1974-present) Adult**

| # | Search History |
| --- | --- |
| 1 | "Attention Deficit and Disruptive Behavior Disorders"/ or Attention Deficit Disorder with Hyperactivity/ |
| 2 | ((attenti* or disrupt*) adj3 (adolescent* or adult* or behav* or child* or class or classes or classroom* or condition* or difficult* or disorder* or learn* or people or person* or poor or problem* or process* or youngster*)).ti. |
| 3 | ((attenti* or disrupt*) adj3 disorder*).ab. |
| 4 | (adhd or addh or ad hd or ad??hd).ti,ab. |
| 5 | (attenti* adj3 deficit*).ti,ab. |
| 6 | (((hyperkin* or hyper kin*) adj1 (syndrome* or disorder*)) or hkd).ti,ab. |
| 7 | (minimal brain adj2 (dysfunct* or disorder*)).ti,ab. |
| 8 | 1 or 2 or 3 or 4 or 5 or 6 or 7 |
| 9 | Mobile Applications/ |
| 10 | exp Internet/ |
| 11 | exp Cell Phone/ |
| 12 | exp Computers, Handheld/ |
| 13 | Medical Informatics Applications/ |
| 14 | Therapy, Computer-Assisted/ |
| 15 | (app or apps).ti,ab. |
| 16 | (online or web or internet or digital*).ti. |
| 17 | ((online or web or internet or digital*) adj3 (based or application* or intervention* or program* or therap*)).ab. |
| 18 | (phone* or telephone* or smartphone* or cellphone* or smartwatch*).ti. |
| 19 | ((phone* or telephone* or smartphone* or cellphone* or smartwatch*) adj3 (based or application* or intervention* or program* or therap*)).ab. |
| 20 | (mobile health or mhealth or m-health or ehealth or e-health or emental or e-mental).ti. |
| 21 | ((mobile health or mhealth or m-health or ehealth or e-health or emental or e-mental) adj3 (based or application* or intervention* or program* or therap*)).ab. |
| 22 | (mobile* adj3 (based or application* or intervention* or device* or technolog*)).ti,ab. |
| 23 | or/9-22 |
| 24 | exp Behavior Therapy/ |
| 25 | exp *cognitive therapy/ |
| 26 | *mindfulness/ |
| 27 | (therap* or psychotherap*).ti,ab. |
| 28 | (cbt or mindful*).ti,ab. |
| 29 | ((cognitive or cognition or behavio?r*) adj2 (therap* or psychotherap*)).ti,ab. |
| 30 | (psych* adj2 (therap* or treatment*)).ti,ab. |
| 31 | "psychoeducation".ti,ab. |
| 32 | adaptation, psychological/ or feedback, psychological/ |
| 33 | feedback, psychological/ or psychosocial intervention/ or psychotherapy, brief/ |
| 34 | or/24-33 |
| 35 | exp exercise/ |
| 36 | exp exercise therapy/ |
| 37 | exp "physical education and training"/ |
| 38 | (pilates or yoga or mckenzie or feldenkrais or swim* or walk* or run* or jog* or treadmill* or tread mill*).ti,ab. |
| 39 | (aerobic* adj (exercise* or train* or therap*)).ti,ab. |
| 40 | ((corrective* or biomechanic*) adj (exercise* or train* or therap*)).ti,ab. |
| 41 | (stretch* adj3 (active* or passive* or relax* or static* or dynamic* or gentl* or ballistic* or force* or isometric or technique* or exercis* or therap*)).ti,ab. |
| 42 | ((strength* or stabil* or program* or train* or therap* or technique* or treat*) adj3 exercise*).ti,ab. |
| 43 | (fitness* adj3 (prog! ram* or t! rain* or therap*)).ti,ab. |
| 44 | (tai ji or tai chi or taichi or taiji or taijiquan).ti,ab. |
| 45 | (qigong or ch'i k#ng or ch'i g#ng or chi k#ng or chi g#ng or qi k#ng or qi g#ng).ti,ab. |
| 46 | yoga/ |
| 47 | exercise movement techniques.mp. [mp=title, book title, abstract, original title, name of substance word, subject heading word, floating sub-heading word, keyword heading word, organism supplementary concept word, protocol supplementary concept word, rare disease supplementary concept word, unique identifier, synonyms, population supplementary concept word, anatomy supplementary concept word] |
| 48 | Life Style/ |
| 49 | or/35-48 |
| 50 | Adult/ |
| 51 | adult.ti,ab. |
| 52 | or/50-51 |
| 53 | (assign* or allocat* or volunteer* or placebo*).ti,ab. |
| 54 | exp controlled clinical trials as topic/ or feasibility studies/ or pilot projects/ |
| 55 | controlled clinical trial.pt. |
| 56 | randomi#ed.ab. |
| 57 | trial.ab. |
| 58 | groups.ab. |
| 59 | or/53-58 |
| 60 | Self-Help Groups/ |
| 61 | support* group*.tw. |
| 62 | group* support*.tw. |
| 63 | peer* support*.tw. |
| 64 | self help group*.tw. |
| 65 | (therapeutic adj social club*).tw. |
| 66 | ((nonmedical or non professional* or lay or layperson* or peer* or voluntary or patient) adj2 (instructor* or tutor* or educator* or consultant* or leader* or expert* or advisor* or facilitat* or deliver* or mentor* or led or guide* or aide* or run)).tw. |
| 67 | or/60-66 |
| 68 | 23 or 34 or 49 or 67 |
| 69 | 8 and 52 and 59 and 68 |

**PsycInfo (1806-present) Adult**

| \| # \| Search History \| \| --- \| --- \| \| 1 \| Attention Deficit Disorder with Hyperactivity/ \| \| 2 \| ((attenti* or disrupt*) adj3 (adolescent* or adult* or behav* or child* or class or classes or classroom* or condition* or difficult* or disorder* or learn* or people or person* or poor or problem* or process* or youngster*)).ti. \| \| 3 \| ((attenti* or disrupt*) adj3 disorder*).ab. \| \| 4 \| (adhd or addh or ad hd or ad??hd).ti,ab. \| \| 5 \| (attenti* adj3 deficit*).ti,ab. \| \| 6 \| (((hyperkin* or hyper kin*) adj1 (syndrome* or disorder*)) or hkd).ti,ab. \| \| 7 \| (minimal brain adj2 (dysfunct* or disorder*)).ti,ab. \| \| 8 \| or/1-7 \| \| 9 \| Mobile Applications/ \| \| 10 \| exp Internet/ \| \| 11 \| exp Mobile Phones/ \| \| 12 \| exp Computers/ \| \| 13 \| exp Digital Interventions/ \| \| 14 \| (app or apps).ti,ab. \| \| 15 \| (online or web or internet or digital*).ti. \| \| 16 \| ((online or web or internet or digital*) adj3 (based or application* or intervention* or program* or therap*)).ab. \| \| 17 \| (phone* or telephone* or smartphone* or cellphone* or smartwatch*).ti. \| \| 18 \| ((phone* or telephone* or smartphone* or cellphone* or smartwatch*) adj3 (based or application* or intervention* or program* or therap*)).ab. \| \| 19 \| (mobile health or mhealth or m-health or ehealth or e-health or emental or e-mental).ti. \| \| 20 \| ((mobile health or mhealth or m-health or ehealth or e-health or emental or e-mental) adj3 (based or application* or intervention* or program* or therap*)).ab. \| \| 21 \| (mobile* adj3 (based or application* or intervention* or device* or technolog*)).ti,ab. \| \| 22 \| or/9-21 \| \| 23 \| exp Behavior Therapy/ \| \| 24 \| exp Cognitive Behavior Therapy/ \| \| 25 \| exp Dialectical Behavior Therapy/ \| \| 26 \| exp Biopsychosocial Approach/ \| \| 27 \| exp Mindfulness/ \| \| 28 \| exp Meditation/ \| \| 29 \| exp Mindfulness-based interventions/ \| \| 30 \| (therap* or psychotherap*).ti,ab. \| \| 31 \| (cbt or mindful*).ti,ab. \| \| 32 \| ((cognitive or cognition or behavio?r*) adj2 (therap* or psychotherap*)).ti,ab. \| \| 33 \| (psych* adj2 (therap* or treatment*)).ti,ab. \| \| 34 \| exp Psychoeducation/ \| \| 35 \| exp Clinical Psychology/ \| \| 36 \| exp Biopsychosocial Approach/ \| \| 37 \| exp Psychosocial Rehabilitation/ \| \| 38 \| exp Interdisciplinary Treatment Approach/ \| \| 39 \| or/23-38 \| \| 40 \| exp exercise/ \| \| 41 \| exp Physical Education/ \| \| 42 \| (pilates or yoga or mckenzie or feldenkrais or swim* or walk* or run* or jog* or treadmill* or tread mill*).ti,ab. \| \| 43 \| (aerobic* adj (exercise* or train* or therap*)).ti,ab. \| \| 44 \| ((corrective* or biomechanic*) adj (exercise* or train* or therap*)).ti,ab. \| \| 45 \| (stretch* adj3 (active* or passive* or relax* or static* or dynamic* or gentl* or ballistic* or force* or isometric or technique* or exercis* or therap*)).ti,ab. \| \| 46 \| ((strength* or stabil* or program* or train* or therap* or technique* or treat*) adj3 exercise*).ti,ab. \| \| 47 \| (fitness* adj3 (program* or train* or therap*)).ti,ab. \| \| 48 \| (tai ji or tai chi or taichi or taiji or taijiquan).ti,ab. \| \| 49 \| (qigong or ch'i k#ng or ch'i g#ng or chi k#ng or chi g#ng or qi k#ng or qi g#ng).ti,ab. \| \| 50 \| yoga/ \| \| 51 \| exercise movement techniques.mp. [mp=title, abstract, heading word, table of contents, key concepts, original title, tests & measures, mesh word] \| \| 52 \| Lifestyle/ \| \| 53 \| exp Lifestyle Changes/ \| \| 54 \| or/40-53 \| \| 55 \| peer* support*.tw. \| \| 56 \| Peer Counseling/ \| \| 57 \| self help group*.tw. \| \| 58 \| (therapeutic adj social club*).tw. \| \| 59 \| ((non medical or non professional* or lay or layperson* or peer* or voluntary or patient) adj2 (instructor* or tutor* or educator* or consultant* or leader* or expert* or advisor* or facilitat* or deliver* or mentor* or led or guide* or aide* or run)).tw. \| \| 60 \| Or/55-59 \| \| 61 \| Middle Adulthood/ \| \| 62 \| Emerging Adulthood/ \| \| 63 \| adult.ti,ab. \| \| 64 \| Older Adulthood/ \| \| 65 \| Or/61-64 \| \| 66 \| (assign* or allocat* or volunteer* or placebo*).ti,ab. \| \| 67 \| exp Clinical Trials/ \| \| 68 \| Randomised Clinical Trials/ \| \| 60 \| Treatment Effectiveness Evaluation/ \| \| 70 \| (pilot or feasibility).ti,ab. \| \| 71 \| Followup Studies/ \| \| 72 \| randomi#ed.ab. \| \| 73 \| trial.ab. \| \| 74 \| groups.ab. \| \| 75 \| Or/66-74 \| \| 76 \| 22 or 39 or 54 or 60 \| \| 77 \| 8 and 65 and 75 and 76 \| |
| --- | --- | --- | --- | --- | --- | --- | --- | --- | --- | --- | --- | --- | --- | --- | --- | --- | --- | --- | --- | --- | --- | --- | --- | --- | --- | --- | --- | --- | --- | --- | --- | --- | --- | --- | --- | --- | --- | --- | --- | --- | --- | --- | --- | --- | --- | --- | --- | --- | --- | --- | --- | --- | --- | --- | --- | --- | --- | --- | --- | --- | --- | --- | --- | --- | --- | --- | --- | --- | --- | --- | --- | --- | --- | --- | --- | --- | --- | --- | --- | --- | --- | --- | --- | --- | --- | --- | --- | --- | --- | --- | --- | --- | --- | --- | --- | --- | --- | --- | --- | --- | --- | --- | --- | --- | --- | --- | --- | --- | --- | --- | --- | --- | --- | --- | --- | --- | --- | --- | --- | --- | --- | --- | --- | --- | --- | --- | --- | --- | --- | --- | --- | --- | --- | --- | --- | --- | --- | --- | --- | --- | --- | --- | --- | --- | --- | --- | --- | --- | --- | --- | --- | --- | --- | --- | --- | --- |

**PsycInfo (1806-present) Adolescent**

| 1. | Attention Deficit Disorder with Hyperactivity/ |
| --- | --- |
| 2. | ((attenti* or disrupt*) adj3 (adolescent* or adult* or behav* or child* or class or classes or classroom* or condition* or difficult* or disorder* or learn* or people or person* or poor or problem* or process* or youngster*)).ti. |
| 3. | ((attenti* or disrupt*) adj3 disorder*).ab. |
| 4. | (adhd or addh or ad hd or ad??hd).ti,ab. |
| 5. | (attenti* adj3 deficit*).ti,ab. |
| 6. | (((hyperkin* or hyper kin*) adj1 (syndrome* or disorder*)) or hkd).ti,ab. |
| 7. | (minimal brain adj2 (dysfunct* or disorder*)).ti,ab. |
| 8. | or/1-7 |
| 9. | Mobile Applications/ |
| 10. | exp Internet/ |
| 11. | exp Mobile Phones/ |
| 12. | exp Computers/ |
| 13. | exp Digital Interventions/ |
| 14. | (app or apps).ti,ab. |
| 15. | (online or web or internet or digital*).ti. |
| 16. | ((online or web or internet or digital*) adj3 (based or application* or intervention* or program* or therap*)).ab. |
| 17. | (phone* or telephone* or smartphone* or cellphone* or smartwatch*).ti. |
| 18. | ((phone* or telephone* or smartphone* or cellphone* or smartwatch*) adj3 (based or application* or intervention* or program* or therap*)).ab. |
| 19. | (mobile health or mhealth or m-health or ehealth or e-health or emental or e-mental).ti. |
| 20. | ((mobile health or mhealth or m-health or ehealth or e-health or emental or e-mental) adj3 (based or application* or intervention* or program* or therap*)).ab. |
| 21. | (mobile* adj3 (based or application* or intervention* or device* or technolog*)).ti,ab. |
| 22. | or/9-21 |
| 23. | exp Behavior Therapy/ |
| 24. | exp Cognitive Behavior Therapy/ |
| 25. | exp Dialectical Behavior Therapy/ |
| 26. | exp Biopsychosocial Approach/ |
| 27. | exp Mindfulness/ |
| 28. | exp Meditation/ |
| 29. | exp Mindfulness-based interventions/ |
| 30. | (therap* or psychotherap*).ti,ab. |
| 31. | (cbt or mindful*).ti,ab. |
| 32. | ((cognitive or cognition or behavio?r*) adj2 (therap* or psychotherap*)).ti,ab. |
| 33. | (psych* adj2 (therap* or treatment*)).ti,ab. |
| 34. | exp Psychoeducation/ |
| 35. | exp Clinical Psychology/ |
| 36. | exp Biopsychosocial Approach/ |
| 37. | exp Psychosocial Rehabilitation/ |
| 38. | exp Interdisciplinary Treatment Approach/ |
| 39. | or/23-38 |
| 40. | exp exercise/ |
| 41. | exp Physical Education/ |
| 42. | (pilates or yoga or mckenzie or feldenkrais or swim* or walk* or run* or jog* or treadmill* or tread mill*).ti,ab. |
| 43. | (aerobic* adj (exercise* or train* or therap*)).ti,ab. |
| 44. | ((corrective* or biomechanic*) adj (exercise* or train* or therap*)).ti,ab. |
| 45. | (stretch* adj3 (active* or passive* or relax* or static* or dynamic* or gentl* or ballistic* or force* or isometric or technique* or exercis* or therap*)).ti,ab. |
| 46. | ((strength* or stabil* or program* or train* or therap* or technique* or treat*) adj3 exercise*).ti,ab. |
| 47. | (fitness* adj3 (program* or train* or therap*)).ti,ab. |
| 48. | (tai ji or tai chi or taichi or taiji or taijiquan).ti,ab. |
| 49. | (qigong or ch'i k#ng or ch'i g#ng or chi k#ng or chi g#ng or qi k#ng or qi g#ng).ti,ab. |
| 50. | yoga/ |
| 51. | exercise movement techniques.mp. [mp=title, abstract, heading word, table of contents, key concepts, original title, tests & measures, mesh word] |
| 52. | Lifestyle/ |
| 53. | exp Lifestyle Changes/ |
| 54. | or/40-53 |
| 55. | Support Groups/ |
| 56. | Social Group Work/ |
| 57. | peer* support*.tw. |
| 58. | Peer Counseling/ |
| 59. | self help group*.tw. |
| 60. | (therapeutic adj social club*).tw. |
| 61. | ((non medical or non professional* or lay or layperson* or peer* or voluntary or patient) adj2 (instructor* or tutor* or educator* or consultant* or leader* or expert* or advisor* or facilitat* or deliver* or mentor* or led or guide* or aide* or run)).tw. |
| 62. | or/55-61 |
| 63. | 22 or 39 or 54 or 62 |
| 64. | (adolescen* or teen*).ti,ab. or "200".ag. or "320".ag. |
| 65. | (assign* or allocat* or volunteer* or placebo*).ti,ab. |
| 66. | exp Clinical Trials/ |
| 67. | exp Intervention/ |
| 68. | Treatment Effectiveness Evaluation/ |
| 69. | (pilot or feasibility).ti,ab. |
| 70. | Followup Studies/ |
| 71. | randomi#ed.ab. |
| 72. | or/65-71 |
| 73. | 8 and 63 and 64 and 72 |

**Medline/Embase Search (Adolescent):**

| 1. | "Attention Deficit and Disruptive Behavior Disorders"/ or Attention Deficit Disorder with Hyperactivity/ |
| --- | --- |
| 2. | ((attenti* or disrupt*) adj3 (adolescent* or adult* or behav* or child* or class or classes or classroom* or condition* or difficult* or disorder* or learn* or people or person* or poor or problem* or process* or youngster*)).ti. |
| 3. | ((attenti* or disrupt*) adj3 disorder*).ab. |
| 4. | (adhd or addh or ad hd or ad??hd).ti,ab. |
| 5. | (attenti* adj3 deficit*).ti,ab. |
| 6. | (((hyperkin* or hyper kin*) adj1 (syndrome* or disorder*)) or hkd).ti,ab. |
| 7. | (minimal brain adj2 (dysfunct* or disorder*)).ti,ab. |
| 8. | 1 or 2 or 3 or 4 or 5 or 6 or 7 |
| 9. | Mobile Applications/ |
| 10. | exp Internet/ |
| 11. | exp Cell Phone/ |
| 12. | exp Computers, Handheld/ |
| 13. | Medical Informatics Applications/ |
| 14. | Therapy, Computer-Assisted/ |
| 15. | (app or apps).ti,ab. |
| 16. | (online or web or internet or digital*).ti. |
| 17. | ((online or web or internet or digital*) adj3 (based or application* or intervention* or program* or therap*)).ab. |
| 18. | (phone* or telephone* or smartphone* or cellphone* or smartwatch*).ti. |
| 19. | ((phone* or telephone* or smartphone* or cellphone* or smartwatch*) adj3 (based or application* or intervention* or program* or therap*)).ab. |
| 20. | (mobile health or mhealth or m-health or ehealth or e-health or emental or e-mental).ti. |
| 21. | ((mobile health or mhealth or m-health or ehealth or e-health or emental or e-mental) adj3 (based or application* or intervention* or program* or therap*)).ab. |
| 22. | (mobile* adj3 (based or application* or intervention* or device* or technolog*)).ti,ab. |
| 23. | or/9-22 |
| 24. | exp Behavior Therapy/ |
| 25. | exp *cognitive therapy/ |
| 26. | *mindfulness/ |
| 27. | (therap* or psychotherap*).ti,ab. |
| 28. | (cbt or mindful*).ti,ab. |
| 29. | ((cognitive or cognition or behavio?r*) adj2 (therap* or psychotherap*)).ti,ab. |
| 30. | (psych* adj2 (therap* or treatment*)).ti,ab. |
| 31. | "psychoeducation".ti,ab. |
| 32. | adaptation, psychological/ or feedback, psychological/ |
| 33. | feedback, psychological/ or psychosocial intervention/ or psychotherapy, brief/ |
| 34. | or/24-33 |
| 35. | exp exercise/ |
| 36. | exp exercise therapy/ |
| 37. | exp "physical education and training"/ |
| 38. | (pilates or yoga or mckenzie or feldenkrais or swim* or walk* or run* or jog* or treadmill* or tread mill*).ti,ab. |
| 39. | (aerobic* adj (exercise* or train* or therap*)).ti,ab. |
| 40. | ((corrective* or biomechanic*) adj (exercise* or train* or therap*)).ti,ab. |
| 41. | (stretch* adj3 (active* or passive* or relax* or static* or dynamic* or gentl* or ballistic* or force* or isometric or technique* or exercis* or therap*)).ti,ab. |
| 42. | ((strength* or stabil* or program* or train* or therap* or technique* or treat*) adj3 exercise*).ti,ab. |
| 43. | (fitness* adj3 (program* or train* or therap*)).ti,ab. |
| 44. | (tai ji or tai chi or taichi or taiji or taijiquan).ti,ab. |
| 45. | (qigong or ch'i k#ng or ch'i g#ng or chi k#ng or chi g#ng or qi k#ng or qi g#ng).ti,ab. |
| 46. | yoga/ |
| 47. | exercise movement techniques.mp. [mp=title, book title, abstract, original title, name of substance word, subject heading word, floating sub-heading word, keyword heading word, organism supplementary concept word, protocol supplementary concept word, rare disease supplementary concept word, unique identifier, synonyms, population supplementary concept word, anatomy supplementary concept word] |
| 48. | Life Style/ |
| 49. | or/35-48 |
| 50. | Self Help/ |
| 51. | Group Support/ |
| 52. | support* group*.tw. |
| 53. | group* support*.tw. |
| 54. | peer* support*.tw. |
| 55. | self help group*.tw. |
| 56. | (therapeutic adj social club*).tw. |
| 57. | ((nonmedical or non professional* or lay or layperson* or peer* or voluntary or patient) adj2 (instructor* or tutor* or educator* or consultant* or leader* or expert* or advisor* or facilitat* or deliver* or mentor* or led or guide* or aide* or run)).tw. |
| 58. | or/50-57 |
| 59. | 23 or 34 or 49 or 58 |
| 60. | Adolescent/ or (adolescen* or teen*).ti,ab. |
| 61. | (assign* or allocat* or volunteer* or placebo*).ti,ab. |
| 62. | exp controlled clinical trials as topic/ or feasibility studies/ or pilot projects/ |
| 63. | controlled clinical trial.pt. |
| 64. | randomi#ed.ab. |
| 65. | trial.ab. |
| 66. | groups.ab. |
| 67. | or/61-66 |
| 68. | 8 and 59 and 60 and 67 |
